# Hemispheric Asymmetry on the Electroencephalogram during General Anesthesia Responsive to Blood Pressure Manipulations

**DOI:** 10.3390/neurolint14040081

**Published:** 2022-12-07

**Authors:** Bryan T. Nycz, Andrew Chalhoub, Gaurav P. Patel, Cassandra E. Dean, Alexander Papangelou

**Affiliations:** Department of Anesthesiology, School of Medicine, Emory University, Atlanta, GA 30307, USA

**Keywords:** Electroencephalogram during anesthesia, hemispheric asymmetry, ischemia

## Abstract

The electroencephalogram (EEG) has been extensively used to detect ischemia and the need for shunting during carotid endarterectomy. Limited literature exists using EEG data to detect ischemia in other surgeries. This case report depicts a 65-year-old man, with extensive vascular history including complete left carotid occlusion and severe right carotid stenosis, who presented for left first rib resection and left subclavian vein balloon angioplasty. Following induction of general anesthesia, frontal EEG (SedLine; Masimo Corporation, Irvine, CA, USA) demonstrated hemispheric asymmetry, which nearly resolved with vasoactive support. At three distinct periods, discordance reoccurred necessitating a higher mean arterial pressure threshold. This case demonstrates EEG patterns concerning for focal spectrographic ischemia and highlights the potential use of EEG signals to capture hypoperfusion and direct vasoactive therapy.

## 1. Introduction

Various electroencephalography-based monitors have been developed to assist anesthesiologists in targeting appropriate depth of anesthesia [1]. Expanded utilities are being investigated including limiting burst suppression in hopes of reducing post-operative delirium and identifying electroencephalogram signatures of intraoperative nociception [2,3].

Raw electroencephalogram (EEG) signals from multichannel montages are used to detect ischemia and the need for shunting during carotid endarterectomy (CEA), but limited literature exists using EEG data to detect ischemia in other surgeries [4]. Signs of ischemia on the EEG may demonstrate loss of fast background frequencies, low-voltage irregular delta activity, and/or total absence of activity. Slowed frequencies begin to occur at cerebral blood flow (CBF) of 25–35 mL/100 g/min with infarction thresholds occurring at 10–12 mL/100 g/min [5].

Similarly, intraoperative frontal EEG monitoring might be expected to reveal evidence of large territory cerebral ischemia and infarction. Loss of power in higher frequencies and increased power in delta band would be clearly demonstrated in the DSA with the associated color changes. The spectral edge frequency (frequency below which 95% of the EEG power exists) would also dramatically decline due to dominant delta activity, and assuming unilateral hemispheric ischemia, exhibit marked asymmetry. To our knowledge, the following is the first reported case of cerebral hypoperfusion captured by intraoperative frontal EEG for a non-carotid procedure. Written Healthcare Insurance Portability and Accountability Act authorization and consent was obtained.

## 2. Case

This case report depicts a 65-year-old man with history of dialysis-dependent end-stage renal disease, subclavian deep vein thrombus, and pulmonary embolism on anticoagulation. He also suffered from autonomic dysfunction following neck radiation, hypertension, complete left ICA occlusion and severe (70–99%) right ICA stenosis, and a ruptured abdominal aortic aneurysm status-post repair. This patient also had chronic chylous effusions following left upper extremity (LUE) brachiocephalic fistula formation, complicated by LUE swelling requiring venoplasty. The patient presented to the operating room for left first rib resection, left subclavian vein balloon angioplasty, fistulogram, and left chest tube placement.

Pre-operatively, the patient was neurologically intact and hemodynamically stable. His right upper extremity non-invasive blood pressure (BP) was 103/70 mmHg and mean arterial pressure (MAP) was 81 mmHg. Access consisted of two 18-gauge peripheral intravenous lines. Standard American Society of Anesthesiology (ASA) monitors were applied, and induction of general anesthesia with endotracheal intubation was performed with fentanyl, lidocaine, propofol, succinylcholine, rocuronium, and ketamine. After induction, the frontal EEG electrodes (Root with SedLine; Masimo Corporation, Irvine, CA, USA) was placed on the patient’s forehead as per manufacturer recommendations.

**Figure 1:**
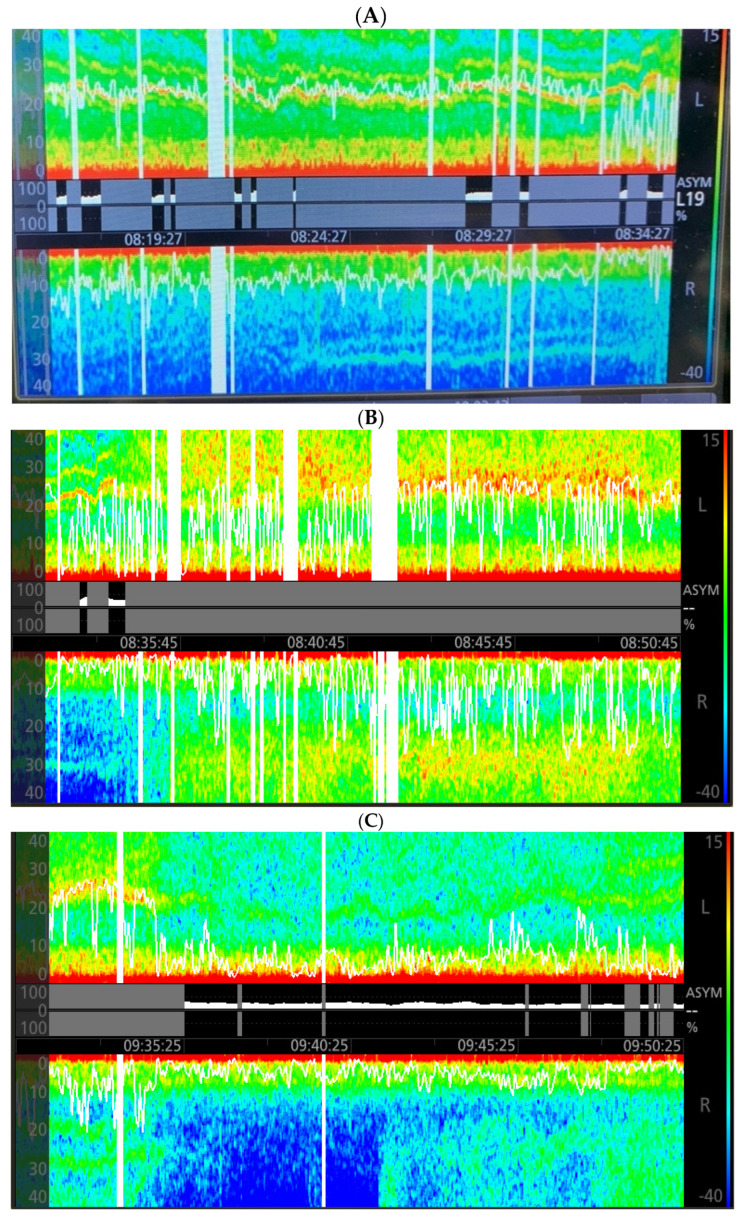

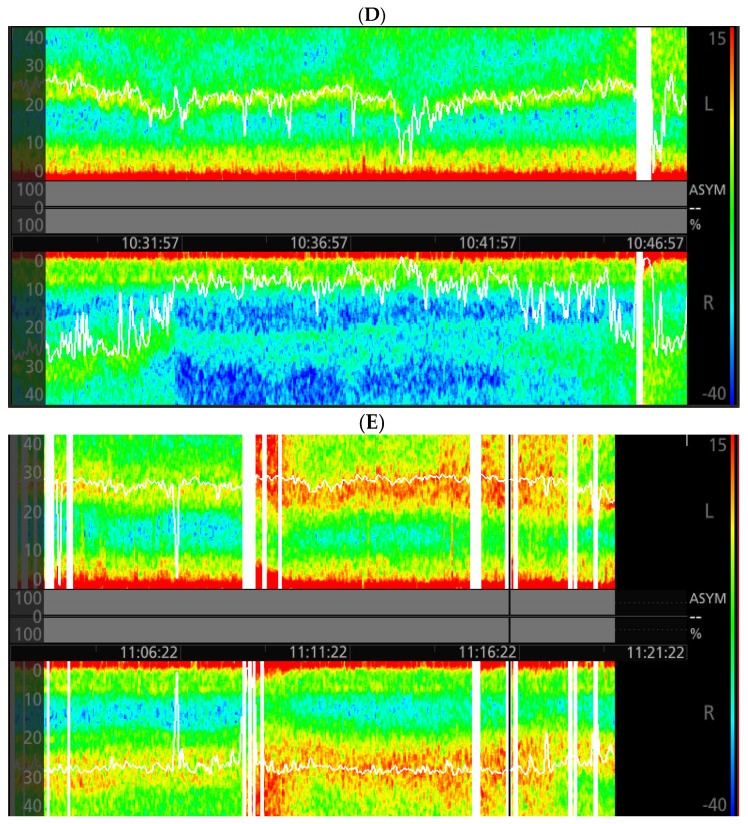
Intraoperative electroencephalogram (EEG) with density spectral array monitoring (Root with SedLine; Masimo Corporation, Irvine, CA, USA) images during general anesthesia with sevoflurane. The top portion of the images represents the spectrogram of the left frontal EEG output and the bottom portion represents the right frontal EEG output in a mirrored orientation. The x-axis is time and y-axis is EEG frequency (hertz). Red colors correspond to EEG frequencies with the highest power and blue represents little to no power with a color gradient defined on the far right of the image. This gradient is known as the Power Spectrum Range (decibels). The middle of the images depicts the Asymmetry Graph, which quantifies asymmetric brain activity and its relative percent. The white horizontal lines within the spectrograms represent the spectral edge frequency where below 95% of the EEG power exists. (**A**) Post-induction snapshot showing asymmetry between the left and right. The DSA on the right shows loss of higher frequency power. The right spectral edge is also dramatically decreased; (**B**) Addition of norepinephrine (NE) to maintain mean arterial pressures (MAP) greater than 95 mmHg results in symmetry in the DSA and spectral edge; (**C**) Reduction in vasoactive support with MAPs dropping to a nadir of 65 mmHg reveals a similar loss of high frequency power on the right with associated decrease in the spectral edge starting at the time 09:30; (**D**) MAP drop to 75 mmHg again reveals loss of high frequency power on the right and decrease in the spectral edge at the time 10:32; (**E**) Immediately prior to and following extubation reveals symmetry in the DSA and spectral edge.

After induction of general anesthesia, ongoing hypotension (MAPs of 50–60 mmHg) ensued, requiring multiple boluses of 16 to 32 mcg of norepinephrine (NE). A right femoral arterial line was placed due to difficult bilateral upper extremity arterial access. During line placement, the non-invasive MAPs ranged from 50–80 mmHg, and significant EEG asymmetry was noted concerning for cerebral ischemia. The right spectral edge measured 10 Hz, while the left measured 25 Hz with a warm band between 20–30 Hz likely reflecting ketamine dosing (Figure 1A). Sevoflurane, the volatile anesthetic used during this case, was age-adjusted and maintained at a mean alveolar concentration of 0.7, guided by the EEG. Due to ongoing intermittent hypotension, a NE infusion was started. When the MAPs were above 95 mmHg, the spectral edge gap narrowed and the DSA appeared nearly symmetric (Figure 1B).

Knowing pre-operative baseline MAP of 81 mmHg, a decision was made to set the lower limit of acceptable MAP to a level greater than 70 mmHg (85% of baseline), allowing for a reduction in the NE infusion rate. Within minutes of MAP reduction, loss of fast activity above 12 Hz was observed in both hemispheres, with the larger effect on the right hemisphere (Figure 1C). The NE infusion rate was increased and spectrographic symmetry returned at MAPs above 95 mmHg. The same pattern in the right hemisphere reoccurred following another drop in MAP (Figure 1D). After these events and with MAPs greater than 95 mmHg, these changes in the right hemisphere did not reoccur (Figure 1E). Neuromuscular blockade was reversed and the patient was extubated on a low dose NE infusion at case end. In the recovery unit, he was weaned from NE and did not exhibit new neurological deficits. Following discontinuation of NE, the patient’s MAPs fluctuated between 76–99 mmHg in the post-operative care unit via arterial monitoring. On six-month clinic follow-up, the patient demonstrated unchanged 70–99% right ICA stenosis, complete left ICA occlusion with collateral flow, and bilateral vertebral anterograde flow.

## 3. Discussion

This case illustrates EEG patterns concerning for unexpected focal ischemia in the setting of severe ICA stenosis. The intra-operative EEG directed vasoactive support to target spectral symmetry and guided volatile anesthetic delivery to maintain hypnosis and avoid burst suppression. To our knowledge, this is the first reported case of cerebral hypoperfusion captured by intraoperative frontal EEG for a non-carotid procedure. Prior to vasoactive support, the observed ischemic pattern was consistent with asymmetric low-voltage delta activity and loss of fast background following carotid clamping reported by Sharbrough et al. during CEA [6]. While these patterns are frequently seen with deep levels of hypnosis or in individuals with poor cognitive reserve, spectral asymmetry would not be encountered [1]. Of critical importance, discordance in the spectrogram coincided with decreased systemic pressures and reproducibly corrected with elevated pressures at a constant level of volatile anesthesia.

Baseline MAP (MAP range) is very difficult to define preoperatively. The decision to utilize absolute thresholds or baseline blood pressure and the modality of measurement to limit perioperative morbidity and mortality is a matter of debate [7,8]. Although our patient’s preoperative MAP was 81 mmHg, under general anesthesia with potential changes in the autoregulation curve, its value in guiding management became limited. Cerebral autoregulation can be impaired in the presence of severe ICA stenosis, with improvement after minimizing the degree of stenosis [9]. Patients with impaired cerebral autoregulation have a higher likelihood of cerebral blood flow (CBF) dropping to the ischemic threshold as blood pressure falls [5]. Conceptually, exhausted cerebral vascular reactivity resulting in intracerebral steal, a paradoxical vasodilatory phenomenon that reroutes CBF to healthy regions and reduces flow to compromised areas, may have contributed to our observations. Hartkamp et al. investigated patients with severe, symptomatic ICA stenosis following administration of vasodilatory pharmacotherapy. They demonstrated steal in the hemisphere ipsilateral to the diseased ICA along with impaired collateral blood flow [10]. We must also mention the possible contribution of increased venous pressure caused by surgical manipulation of the SVC, which would further reduce cerebral perfusion pressure.

DSA asymmetry was pronounced at the beginning of the case, during which time significant hypotension was combined with ketamine effect. The inability of the hypoperfused hemisphere to augment cortical activity is certainly extremely important, as it could have very likely led to post-operative issues such as delirium or other cognitive issues [7].

This case demonstrates that while MAP can certainly be used to guide hemodynamic management, it alone cannot always be trusted. Impaired cerebral autoregulation, those with severe ICA stenosis, and a variety of other factors under general anesthesia may make gauging cerebral blood flow difficult [5,6,7,8,9,10]. As such, EEG monitoring may allow another avenue for preventing cerebral ischemia. However, limitations do exist. A variety of medications (ketamine for example as noted above) can cause EEG related changes. Furthermore, EEG interpretation is not currently a core skillset for anesthesiologists to learn during training. As such, its utility may be limited until further training becomes mainstream.

Rapid detection and intervention are paramount to prevent focal or global tissue ischemia possibly leading to infarction. While a variety of vasoactive agents can be used to augment blood pressure, knowing what MAP level is required is difficult. The pharmacologic armamentarium available to the anesthesiologist in a variety of surgical procedures is often limited [11]; as such, utilizing this EEG based approach may improve appropriate management. In this setting, the lower limit MAP goal must be based on beat-to-beat arterial blood pressure targeting EEG symmetry. As the deleterious impact of even mild hypotension is currently being stressed in the anesthesiology world [12], EEG based techniques may be useful on a variety of different surgical cases (including trauma, transplantation, and obstetric anesthesia). More research is certainly needed in this realm, but this case stresses the importance of being able to recognize, interpret, and react to atypical spectrographic patterns to minimize potential poor outcomes.

## Data Availability

Not applicable.

## References

[1] Purdon P.L., Sampson A., Pavone K.J., Brown E.N. (2015). Clinical Electroencephalography for Anesthesiologists: Part I: Background and Basic Signatures. Anesthesiology.

[2] Sun Y., Ye F., Wang J., Ai P., Wei C., Wu A., Xie W. (2020). Electroencephalography-Guided Anesthetic Delivery for Preventing Postoperative Delirium in Adults: An Updated Meta-analysis. Obstet. Anesth. Dig..

[3] García P.S., Kreuzer M., Hight D., Sleigh J.W. (2021). Effects of noxious stimulation on the electroencephalogram during general anaesthesia: A narrative review and approach to analgesic titration. Br. J. Anaesth..

[4] Laman D.M., Wieneke G.H., Van Duijn H., Veldhuizen R.J., Van Huffelen A.C. (2005). QEEG Changes During Carotid Clamping in Carotid Endarterectomy: Spectral Edge Frequency Parameters and Relative Band Power Parameters. J. Clin. Neurophysiol..

[5] Foreman B., Claassen J. (2012). Quantitative EEG for the detection of brain ischemia. Annual Update in Intensive Care and Emergency Medicine 2012.

[6] Sharbrough F.W., Messick J.M., Sundt T.M. (1973). Correlation of continuous electroencephalograms with cerebral blood flow measurements during carotid endarterectomy. Stroke.

[7] Meng L., Yu W., Wang T., Zhang L., Heerdt P.M., Gelb A.W. (2018). Blood Pressure Targets in Perioperative Care. Hypertension.

[8] Saugel B., Reese P.C., Sessler D.I., Burfeindt C., Nicklas J.Y., Pinnschmidt H.O., Reuter D.A., Südfeld S. (2019). Automated Ambulatory Blood Pressure Measurements and Intraoperative Hypotension in Patients Having Noncardiac Surgery with General Anesthesia: A Prospective Observational Study. Anesthesiology.

[9] Tang S.-C., Huang Y.-W., Shieh J.-S., Huang S.-J., Yip P.-K., Jeng J.-S. (2008). Dynamic cerebral autoregulation in carotid stenosis before and after carotid stenting. J. Vasc. Surg..

[10] Hartkamp N.S., Hendrikse J., de Borst G.J., Kappelle L.J., Bokkers R.P. (2018). Intracerebral steal phenomenon in symptomatic carotid artery disease. J. Neuroradiol..

[11] Running K., Weinberg D., Trudo W., Sullivan C.L., Patel G.P. (2021). Intraoperative Use of Angiotensin II for Severe Vasodilatory Shock During Liver Transplantation: A Case Report. A&A Pr..

[12] Saugel B., Sessler D.I. (2020). Perioperative Blood Pressure Management. Anesthesiology.

